# Never forget the basics while seeking correct diagnosis and management

**DOI:** 10.1016/j.clinme.2024.100232

**Published:** 2024-08-14

**Authors:** Ponnusamy Saravanan

**Affiliations:** a Warwick Applied Health, Warwick Medical School, University of Warwick, Coventry, United Kingdom; b Diabetes, Endocrinology and Metabolism, George Eliot Hospital NHS Trust, Nuneaton, United Kingdom

The NHS has a fantastic system for mapping patient care pathways. This means the right care delivered to the right person, at the right time and in the right place. However, with stretched services across the NHS, this important aspect is being challenged, resulting in compromised care. A key contributor to this is the constant changing of commissioning and delivery of care in recent decades. While innovation-based ‘change’ is central to providing high-quality clinical care, not all changes in our care pathway are supported by high-quality evidence. This issue of *Clinical Medicine* highlights two articles that emphasise why this is even more crucial for the NHS today.

Several initiatives have been introduced in the NHS to ease pressure on emergency beds, but the evidence for some, like same-day emergency care (SDEC), is limited. Many NHS trusts have adopted SDEC in various ways, but finding the right patients for this pathway is key to its success in reducing pressure on acute beds without compromising quality of care. A systematic review by Atkin *et al*^1^ aimed to improve patient selection for SDEC, but it highlighted a lack of overall evidence for the programme. The studies were few and heterogeneous, and there was not enough information on how SDEC is actually used in practice. This highlights the urgent need for more research to develop a wider evidence base for SDEC. It also shows the importance of having a clear plan to gather evidence whenever a care pathway is changed in the NHS.

An article by Reynolds and Chhetri^2^ stresses the importance of seeing the right specialist quickly for motor neurone disease (MND). This is because MND can cause serious problems very quickly after diagnosis, with a median survival rate of 24–48 months. It is a reminder that doctors should never forget the art of ‘what are the likely differential diagnoses’ for any presenting complaint(s) – whether in primary care or at the front door of an acute hospital.

## Declaration of competing interests

None.
